# Capsular polysaccharide and lipopolysaccharide O type analysis of *Klebsiella pneumoniae* isolates by genotype in China

**DOI:** 10.1017/S0950268820001788

**Published:** 2020-08-12

**Authors:** Z. Y. Zhang, R. Qin, Y. H. Lu, J. Shen, S. Y. Zhang, C. Y. Wang, Y. Q. Yang, F. P. Hu, P. He

**Affiliations:** 1 Experimental Teaching Center, School of Medicine, Shanghai Jiao Tong University School of Medicine, Shanghai, China; 2 Department of Medical Microbiology and Immunology, Shanghai Jiao Tong University School of Medicine, Shanghai, China; 3 Huashan hospital, Shanghai Medical College, Fudan University, Shanghai, China

**Keywords:** Antibiotic resistance, capsular polysaccharide, genotype, *Klebsiella pneumoniae*, lipopolysaccharide O type

## Abstract

*Klebsiella pneumoniae* is a common pathogen associated with nosocomial infections and is characterised serologically by capsular polysaccharide (K) and lipopolysaccharide O antigens. We surveyed a total of 348 non-duplicate *K. pneumoniae* clinical isolates collected over a 1-year period in a tertiary care hospital, and determined their O and K serotypes by sequencing of the *wbb Y* and *wzi* gene loci, respectively. Isolates were also screened for antimicrobial resistance and hypervirulent phenotypes; 94 (27.0%) were identified as carbapenem-resistant (CRKP) and 110 (31.6%) as hypervirulent (hvKP). isolates fell into 58 K, and six O types, with 92.0% and 94.2% typeability, respectively. The predominant K types were K14K64 (16.38%), K1 (14.66%), K2 (8.05%) and K57 (5.46%), while O1 (46%), O2a (27.9%) and O3 (11.8%) were the most common. CRKP and hvKP strains had different serotype distributions with O2a:K14K64 (41.0%) being the most frequent among CRKP, and O1:K1 (26.4%) and O1:K2 (17.3%) among hvKP strains. Serotyping by gene sequencing proved to be a useful tool to inform the clinical epidemiology of *K. pneumoniae* infections and provides valuable data relevant to vaccine design.

*Klebsiella pneumoniae* (KP) is a common opportunistic pathogen of hospital- and community-acquired infections, resulting in pneumonia, bronchitis, urinary tract and wound infections. The species primarily afflicts patients at the extremes of age who are immunocompromised, have undergone long-term antibiotic therapy and/or with underlying predisposing conditions. The management of these infections is often complicated by the emergence of antimicrobial resistance which can be associated with high morbidity and mortality rates [1]. In recent years, carbapenem-resistant *K. pneumoniae* (CRKP) and hypervirulent strains (hvKP) have become widespread in China and also globally, and pose a significant threat to public health [2].

Several methods have been used for the type identification of *K. pneumoniae* isolates including capsular polysaccharide (K) and lipopolysaccharide (O) antigen characterisation, DNA restriction analysis by pulsed-field gel electrophoresis and multilocus sequence typing, among others [3]. Both O and K serotypes have been associated with virulence, particularly for K1, K2 and K57 hvKP strains [4]. O-serotyping is seldom performed today but limited past surveys report O1, O3 and O2a to be among the most frequent strain types [5].

A comprehensive understanding of the seroepidemiology of clinical isolates can be helpful for the design of vaccines to prevent infection through both active and passive immunisation, as has been shown for other encapsulated pathogens, notably *Streptococcus pneumoniae*. As access to O and K typing antisera is no longer a viable option, and knowledge of serotype distributions among *K. pneumoniae* isolates in mainland China is limited, we undertook a prevalence study of clinical isolates from our tertiary hospital centre in Shanghai using gene sequencing of O and K serotype-specific chromosomal regions to determine the distribution of strain serotypes.

Three hundred and forty-eight *K. pneumoniae* clinical isolates were collected from patients in different departments between March 2018 and October 2019. Bacterial identification and antimicrobial susceptibility testing were performed by the Vitek2 system (Biomérieux, France) and susceptibility results were reported according to Clinical and Laboratory Standards Institute (CLSI) guidelines. A CRKP strain was defined as an isolate resistant to at least one carbapenem antibiotic. The hvKP phenotype was confirmed by the string test as previously described [4]. Capsular (K) antigen type was determined by *wzi* gene sequence alignment [6]. O genotypes were characterised by the sequence of *wb* gene clusters [7] using two sets of primers. The first set detected O1/O2, O3, O4, O5, O8, O9 and O12 alleles at *wzm-wzt* loci, and the other determined O1 and O2ac alleles. Isolates unreactive with the latter O type primers but positive for O1/O2 alleles were considered to be of the O2a genotype.

Of the 348 isolates, 94 were classed as CRKP and 110 as hvKP phenotypes. Patients ³60 years old accounted for 222 (63.8%) of all isolates; the great majority (299) were from hospitalised patients and the remainder from out-patients. The average length of stay for all inpatients was 38.0 ± 75.6 days, while for patients with CRKP, it was 51.3 ± 90.5 days. In contrast, the average length of stay of patients infected with CSKP (32.5 ± 68.0) or hvKP (27.0 ± 42.8) was significantly lower than for those with CRKP (*P* < 0.001). Most patients (81.6%) had an indwelling device and likewise for those (81.0%) with CRKP. The mortality rate for all patients was 11.8%, and higher for those harbouring CRKP (12.8%) and hvKP (13.64%) (*P* < 0.05).

Most isolates originated from respiratory specimens (58.6%), followed by urinary tract (16.7%) and blood (14.7%). The highest proportion of patients were in ICU (29.0%), followed by the emergency department (10.9%), neurosurgery (10.6%), rheumatology (8.0%) and urology (5.7%). Notably, the highest incidence of CRKP infections occurred in neurosurgery (27.3%) and accounted for 70.3% of all specimens from that department. One hundred and sixty-two (46.5%) patients were classified as nosocomial having acquired the infection after 48 h of admission to hospital. No distinction was made between health care-associated and community-acquired infections.

All isolates were resistant to ampicillin, but none was resistant to colistin, tigecycline and polymyxin. Twenty-nine CRKP-infected patients had received carbapenem antibiotics in the 3 months prior to recovery of the isolate. The hvKP isolates were generally susceptible to antibiotics (>80%) compared with CRKP (0–18.1%). CRKP isolates were almost uniformly resistant to antibiotics (>98%) with the exception of amikacin (susceptibility 18.1%) and gentamicin (susceptibility 6.4%).

Serotype data for all isolates are summarised in the Supplementary Table. The great majority (92.1%) were assigned to a K type and their distribution according to phenotype is shown in Figure 1. Fifty-eight distinct K types were identified, the most frequent being K14K64 (16.4%), K1 (14.6%), K2 (8.0%), K57 (5.5%) and K54 (3.7%) (Fig. 1a). Carbapenem-susceptible isolates (*n* = 254) fell into 57 K types with six types (K1, 20.6%; K2, 10.5%; K57 7.1%; K54, 4.6%; K14K64, 4.2%; and K5, 4.20%), accounting for almost half of this phenotype (Fig. 1b). Likewise, the 94 carbapenem-resistant isolates were grouped into 19 capsular types with the most common being K14K64 (50.5%), K19 (9.5%), K47 (6.3%), K60 (6.3%) and K61 (5.3%) (Fig. 1c). The most frequent of 17 K serotypes identified among hvKP isolates were K1 (30.9%), K2 (19.1%), K57 (14.5%), K5 (8.2%) and K14K64 (6.4%) (Fig. 1d).

**Fig. 1.**
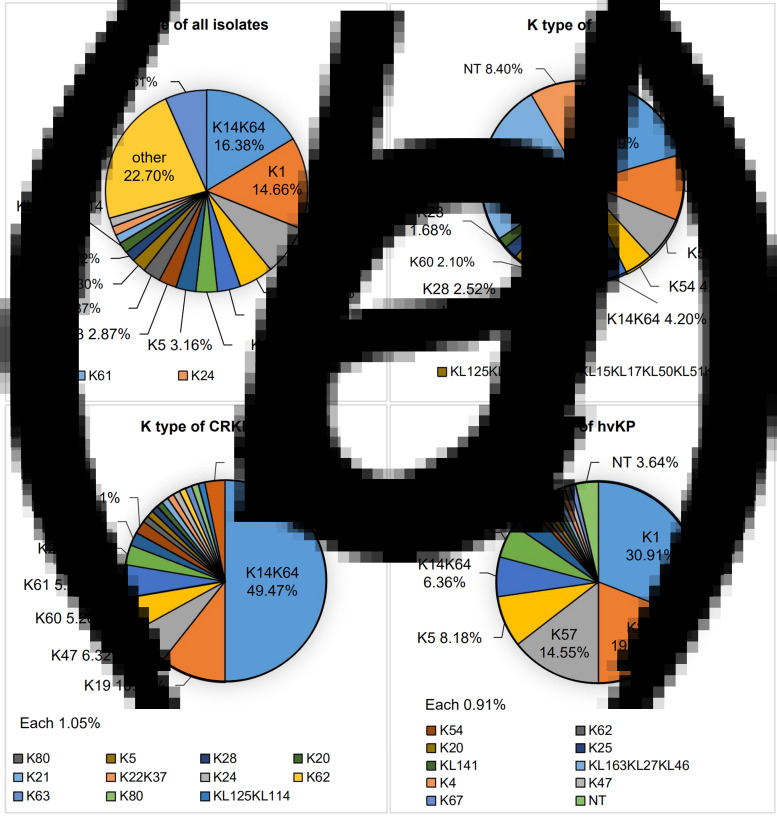
The distribution of K types. (a) The distribution of K types in total 348 isolates. The capsule type of *K. pneumoniae* was determined by wzi gene sequencing. The proportion of K24, K61 and K62 was 1.44%. Forty-three K types with less than five isolates were merged into other. (b) The distribution of K types in 254 isolates of CSKP. The proportion of KL125KL114 and KL15KL17KL50KL51KL52 was 2.10%. The proportion of K24, K25, K62 and KL163KL27KL46 was 1.57%. Forty-four K types with less than five isolates were merged into other. (c) The distribution of K types in 94 isolates of CRKP. The proportion of each K type in the legend was 1.05%. (d) The distribution of K types in 110 isolates of hvKP. The proportion of each K type in the legend was 0.91%. NT, no type.

Six O genotypes (typeability 94.3%) were identified among all isolates with three types (O1, 46.0%; O2a, 27.9%; and O3, 11.8%) together accounting for 85.6% of all isolates. PCR products were not obtained for 20 isolates.

In total, 80 distinct O:K genotype combinations were identified; 15 isolates were ascribed only to an O or K type. Overall sero-typeability was 98.6% with the most common combinations being O2a:K14K64 (*n* = 46), of which 39 and five isolates were CRKP and hvKP, respectively. For the combination O1:K1 (*n* = 45, 29 were hvKP); other frequent combinations were O1:K2 (*n* = 25) and O3:K57 (*n* = 16). The latter two serotype combinations accounted for 19 and 14 hvKP isolates, respectively. Eleven hvKP isolates were carbapenem-resistant, including four of O2a:K14K64, two each of O1:K2 and O3:K57, along with single isolates of O1:K1, O1:K14K64 and O3:K5. Regarding associations of capsular and O serotypes, the majority (80.7%) of K14K64 isolates were O2a, while isolates of K1, K2, K19, K5 and K60 were grouped predominantly in serotype O1. Moreover, serotype O3 was strongly associated with K57 isolates but no association of K54 and K63 isolates with an O type was noted. Lastly, five isolates of K47 were O-non-typeable.

*Klebsiella pneumoniae* has been reported as the second most isolated bacterial pathogen causing opportunist infections in China from 2017 to date [8]. Likewise, the CRKP and hvKP phenotypes are considered to represent the most clinically problematic pathogens worldwide owing to their associated morbidity and mortality.

In this study, a substantial proportion of hospitalised patients were infected with CRKP. Approximately 80% were nosocomially acquired and strongly associated with the use of indwelling medical devices compared with CSKP isolates (88.2% *vs.* 49.2%, respectively). The highest rate of CRKP infections was associated with neurosurgical and ICU departments, the latter having been previously identified as an independent risk factor for acquisition of such strains [9] owing to post-surgical vulnerability, indwelling device use and antimicrobial prophylaxis. Surprisingly, all CRKP infections in the neurosurgery department were hospital-acquired and constituted 70.3% of all patients in the neurosurgery department. The increased risk of nosocomial infection in the neurosurgery department was most likely due to longer term hospitalisation of patients (92.7 ± 184.0) compared with all the other departments (30.2 ± 35.3).

Although hvKP isolates were found to be generally susceptible to antimicrobials, it is noteworthy that 11 of 110 hvKP isolates proved to be carbapenem-resistant and accounted for 3.2% of the total study collection, which alerts us to the emergence of hv-CRKP.

In this survey, serotype K14K64 was most common (16.4%), followed by K1 (14.6%) and K2 (8.0%). Differences in the distribution of K types are most likely due to geographical source. Early serotyping surveys reported K2, K8, K9, K21 and K24 to be among the most frequent [10], but few such surveys have been published. Serotypes K1–K6 are historically more associated with severe respiratory infection and septicaemia in humans [11, 12], while K1, and to a lesser extent K2, has been strongly linked to invasive multisystem disease in SE Asia [4]. K54 (17.1%), K28 (4.1%) and K17 (3.1%) were the most common serotypes in an Australian setting, and K54 was associated with a nosocomial source [13]. Serotype K1 appears to be relatively uncommon in North America and Europe but there is evidence that human K1 isolates from three continents were genetically closely related [14].

Our results showed that the K type distribution among CRKP differed substantially from hvKP isolates with K14K64 (49.5%), K19 (10.5%), K47 (6.3%), K60 (5.3%) and K61 (5.3%) being the most common CRKP serotypes. K64 serotype was also reported to be the major type of CRKP (38%) in Taiwan [15]. In contrast, K47 (66.1%) predominated among isolates from a study in Nanchang, and K64 accounted for only 7.1% in that series [16]. As expected from other surveys of hypervirulent strains, serotypes K1 (30.9%) and K2 (19.1%) and K5 (8.2%) were the most frequent among our hvKP isolates. Both K57 and K5 isolates were associated with liver abscesses. Interestingly, of the seven K14K64 hvKP isolates, five were carbapenem-resistant. Most of O2a:K14K64 (84.8%) and O1:K14K64 (87.8%) strains were CRKP, and most of O1:K2 (76.0%), O3:K57 (87.5%), O1:K1 (64.4%) and O3:K5 (100%, only two strains) were hvKP. The possibility that the hv-CRKP strains may have acquired a virulence or antibiotic resistance-associated plasmid merits further investigation.

The combination of certain O and K serotypes is of interest. Serotype O2a accounted for 80% of K14K64 isolates; O1 was predominantly associated with K1 (88.2%) as well as with K2 (89.3%), and less so with serotypes K19, K5 and K60. Strains of K54 and K63 were evenly distributed in both O types, but notably K47 was linked with strains lacking a demonstrable O type. Despite these associations, O and K combinations may aid the tracking and evolution of strains in outbreak situations if used in conjunction with DNA profiling and sequence-based methods.

A significant limitation of our study is that isolates were collected in a single hospital and so the serotype distribution data might not be representative of the wider regional or national situation. Second, hvKP isolates were identified only by their hypermucoviscosity on agar culture and confirmation of the presence of plasmid genes encoding hypervirulence was not undertaken [17]

In conclusion, molecular typing of O and K antigens of *K. pneumoniae* provided a useful tool to determine serotype distribution in a large collection of isolates from a tertiary hospital setting in China. The predominant K types were K14K64, K1, K2 and K57. Carbapenem-resistant strains exhibited different serotype distributions from those of the hypervirulent phenotype with serotype combination O2a:K14K64 predominating in the former, and O1:K1 and O1:K2 among the latter. These data are of value to inform vaccine design should they be considered as a potential therapeutic approach.

## Data Availability

The authors confirm that the data supporting the findings of this study are available within the article and its Supplementary materials.

## Supplementary material

For supplementary material accompanying this paper visit http://dx.doi.org/10.1017/S0950268820001788. click here to view supplementary material
