# Supplementary material for: Capsular polysaccharide and lipopolysaccharide O type analysis of Klebsiella pneumoniae isolates by genotype in China
Source: Epidemiol Infect. 2020 Aug 12;148:e191. doi: 10.1017/S0950268820001788 (PMC7488366; doi:10.1017/S0950268820001788)
Supplement: Supplementary file 1 [file S0950268820001788sup001.docx]

**Epidemiology and Infection**

**Capsular polysaccharide and lipopolysaccharide O type analysis of** ***Klebsiella pneumoniae* isolates by genotype in China**

**ZY. Zhang^1†^, R. Qin^1†^, YH. Lu^1^, J. Shen^1^, SY. Zhang^1^, CY. Wang^1^, YQ. Yang^1^, FP. Hu^2*^, P. He^1*^**

**Supplementary Material**

| **Supplement Table S1 O:K types in total，CRKP and hvKP isolates** | | | | | | | | |
| --- | --- | --- | --- | --- | --- | --- | --- | --- |
| O: K type | Total | | CRKP | | CSKP | | hvKP | |
| O2a:K14K64 | 46 | 13.22% | 39 | 41.05% | 7 | 2.76% | 5 | 4.55% |
| O1:K1 | 45 | 12.93% | 2 | 2.11% | 43 | 16.93% | 29 | 26.36% |
| O1:K2 | 25 | 7.18% | 2 | 2.11% | 23 | 9.06% | 19 | 17.27% |
| O3:K57 | 16 | 4.60% | 2 | 2.11% | 14 | 5.51% | 14 | 12.73% |
| O1:K19 | 11 | 3.16% | 9 | 9.47% | 2 | 0.79% |  |  |
| O1:K14K64 | 11 | 3.16% | 8 | 8.42% | 3 | 1.18% | 2 | 1.82% |
| O1:K60 | 9 | 2.59% | 5 | 5.26% | 4 | 1.57% |  |  |
| O1:K5 | 8 | 2.30% |  |  | 8 | 3.15% | 6 | 5.45% |
| O2a:K54 | 7 | 2.01% |  |  | 7 | 2.76% | 1 | 0.91% |
| O5:K63 | 6 | 1.72% |  |  | 6 | 2.36% | 4 | 3.64% |
| O1:K54 | 6 | 1.72% | 2 | 2.11% | 4 | 1.57% |  |  |
| O5:NT | 5 | 1.44% |  |  | 5 | 1.97% | 3 | 2.73% |
| O4:KL15KL17KL50KL51KL52 | 5 | 1.44% |  |  | 5 | 1.97% |  |  |
| O2a:K61 | 5 | 1.44% | 5 | 5.26% |  | 0.00% |  |  |
| O1:NT | 5 | 1.44% | 1 | 1.05% | 4 | 1.57% | 1 | 0.91% |
| NT:NT | 5 | 1.44% | 1 | 1.05% | 4 | 1.57% |  |  |
| NT:K47 | 5 | 1.44% | 4 | 4.21% | 1 | 0.39% | 1 | 0.91% |
| O5:KL125KL114 | 4 | 1.15% |  |  | 4 | 1.57% |  |  |
| O3:NT | 4 | 1.15% | 1 | 1.05% | 3 | 1.18% |  |  |
| O2a:K62 | 4 | 1.15% | 1 | 1.05% | 3 | 1.18% | 1 | 0.91% |
| O2a:K20 | 4 | 1.15% | 1 | 1.05% | 3 | 1.18% | 1 | 0.91% |
| O2a:K1 | 4 | 1.15% |  |  | 4 | 1.57% | 3 | 2.73% |
| O1:K63 | 4 | 1.15% | 1 | 1.05% | 3 | 1.18% | 2 | 1.82% |
| O1:K24 | 4 | 1.15% | 1 | 1.05% | 3 | 1.18% |  |  |
| O1:K22K37 | 4 | 1.15% | 1 | 1.05% | 3 | 1.18% |  |  |
| O2a:K57 | 3 | 0.86% |  |  | 3 | 1.18% | 2 | 1.82% |
| O2a:K28 | 3 | 0.86% |  |  | 3 | 1.18% |  |  |
| O1:K28 | 3 | 0.86% |  |  | 3 | 1.18% |  |  |
| O1:K25 | 3 | 0.86% |  |  | 3 | 1.18% |  |  |
| O1:K23 | 3 | 0.86% |  |  | 3 | 1.18% | 3 | 2.73% |
| O3:KL163KL27KL46 | 2 | 0.57% |  |  | 2 | 0.79% | 1 | 0.91% |
| O3:K5 | 2 | 0.57% | 1 | 1.05% | 1 | 0.39% | 2 | 1.82% |
| O3:K31 | 2 | 0.57% |  |  | 2 | 0.79% |  |  |
| O3:K14 | 2 | 0.57% |  |  | 2 | 0.79% |  |  |
| O3:K10 | 2 | 0.57% |  |  | 2 | 0.79% |  |  |
| O3:K1 | 2 | 0.57% |  |  | 2 | 0.79% | 2 | 1.82% |
| O2a:NT | 2 | 0.57% |  |  | 2 | 0.79% |  |  |
| O2a:KL139 | 2 | 0.57% |  |  | 2 | 0.79% |  |  |
| O2a:K80 | 2 | 0.57% | 1 | 1.05% | 1 | 0.39% |  |  |
| O2a:K2 | 2 | 0.57% |  |  | 2 | 0.79% | 2 | 1.82% |
| O12:NT | 2 | 0.57% |  |  | 2 | 0.79% |  |  |
| O1:K47 | 2 | 0.57% | 1 | 1.05% | 1 | 0.39% |  |  |
| O1:K39 | 2 | 0.57% |  |  | 2 | 0.79% |  |  |
| O1:K16 | 2 | 0.57% |  |  | 2 | 0.79% |  |  |
| NT:KL163KL27KL46 | 2 | 0.57% |  |  | 2 | 0.79% |  |  |
| O5:KL137 | 1 | 0.29% |  |  | 1 | 0.39% |  |  |
| O5:K60 | 1 | 0.29% |  |  | 1 | 0.39% |  |  |
| O5:K16 | 1 | 0.29% |  |  | 1 | 0.39% |  |  |
| O4:KL141 | 1 | 0.29% |  |  | 1 | 0.39% | 1 | 0.91% |
| O4:KL102KL149KL155 | 1 | 0.29% |  |  | 1 | 0.39% |  |  |
| O4:K81 | 1 | 0.29% |  |  | 1 | 0.39% |  |  |
| O4:K36 | 1 | 0.29% |  |  | 1 | 0.39% |  |  |
| O3:KL141 | 1 | 0.29% |  |  | 1 | 0.39% |  |  |
| O3:KL125KL114 | 1 | 0.29% |  |  | 1 | 0.39% |  |  |
| O3:KL123 | 1 | 0.29% |  |  | 1 | 0.39% |  |  |
| O3:KL110 | 1 | 0.29% |  |  | 1 | 0.39% |  |  |
| O3:KL105 | 1 | 0.29% |  |  | 1 | 0.39% |  |  |
| O3:K39 | 1 | 0.29% |  |  | 1 | 0.39% |  |  |
| O3:K38 | 1 | 0.29% |  |  | 1 | 0.39% |  |  |
| O3:K30 | 1 | 0.29% |  |  | 1 | 0.39% |  |  |
| O3:K25 | 1 | 0.29% |  |  | 1 | 0.39% | 1 | 0.91% |
| O2a:KL140 | 1 | 0.29% |  |  | 1 | 0.39% |  |  |
| O2a:KL136 | 1 | 0.29% |  |  | 1 | 0.39% |  |  |
| O2a:KL102KL149KL155 | 1 | 0.29% |  |  | 1 | 0.39% |  |  |
| O2a:K5 | 1 | 0.29% |  |  | 1 | 0.39% | 1 | 0.91% |
| O2a:K47 | 1 | 0.29% | 1 | 1.05% |  |  |  |  |
| O2a:K41 | 1 | 0.29% |  |  | 1 | 0.39% |  |  |
| O2a:K4 | 1 | 0.29% |  |  | 1 | 0.39% | 1 | 0.91% |
| O2a:K35 | 1 | 0.29% |  |  | 1 | 0.39% |  |  |
| O2a:K3 | 1 | 0.29% |  |  | 1 | 0.39% |  |  |
| O2a:K27 | 1 | 0.29% |  |  | 1 | 0.39% |  |  |
| O2a:K24 | 1 | 0.29% |  |  | 1 | 0.39% |  |  |
| O2a:K23 | 1 | 0.29% |  |  | 1 | 0.39% | 1 | 0.91% |
| O2a:K21 | 1 | 0.29% | 1 | 1.05% |  |  |  |  |
| O12:K33 | 1 | 0.29% |  |  | 1 | 0.39% |  |  |
| O1:KL56KL63KL114KL148 | 1 | 0.29% |  |  | 1 | 0.39% |  |  |
| O1:KL140 | 1 | 0.29% |  |  | 1 | 0.39% |  |  |
| O1:KL125KL114 | 1 | 0.29% | 1 | 1.05% |  |  |  |  |
| O1:KL116 | 1 | 0.29% |  |  | 1 | 0.39% |  |  |
| O1:KL110 | 1 | 0.29% |  |  | 1 | 0.39% |  |  |
| O1:KL108 | 1 | 0.29% |  |  | 1 | 0.39% |  |  |
| O1:KL102KL149KL155 | 1 | 0.29% |  |  | 1 | 0.39% |  |  |
| O1:K8 | 1 | 0.29% |  |  | 1 | 0.39% |  |  |
| O1:K62 | 1 | 0.29% |  |  | 1 | 0.39% |  |  |
| O1:K55 | 1 | 0.29% |  |  | 1 | 0.39% |  |  |
| O1:K41 | 1 | 0.29% |  |  | 1 | 0.39% |  |  |
| O1:K21 | 1 | 0.29% |  |  | 1 | 0.39% |  |  |
| O1:K17 | 1 | 0.29% |  |  | 1 | 0.39% |  |  |
| NT:KL151 | 1 | 0.29% |  |  | 1 | 0.39% |  |  |
| NT:KL132 | 1 | 0.29% |  |  | 1 | 0.39% |  |  |
| NT:K9 | 1 | 0.29% |  |  | 1 | 0.39% |  |  |
| NT:K81 | 1 | 0.29% |  |  | 1 | 0.39% |  |  |
| NT:K80 | 1 | 0.29% |  |  | 1 | 0.39% |  |  |
| NT:K67 | 1 | 0.29% |  |  | 1 | 0.39% | 1 | 0.91% |
| NT:K2 | 1 | 0.29% | 1 | 1.05% |  |  |  |  |
| NT:K19 | 1 | 0.29% | 1 | 1.05% |  |  |  |  |
